# Post-Harvest Use of Ultraviolet Light (UV) and Light Emitting Diode (LED) to Enhance Bioactive Compounds in Refrigerated Tomatoes

**DOI:** 10.3390/molecules26071847

**Published:** 2021-03-25

**Authors:** Nieves Baenas, Celia Iniesta, Rocío González-Barrio, Vanesa Nuñez-Gómez, María Jesús Periago, Francisco Javier García-Alonso

**Affiliations:** Department of Food Technology, Food Science and Nutrition, Faculty of Veterinary Sciences, Regional Campus of International Excellence “Campus Mare-Nostrum”, University of Murcia, Campus de Espinardo, 30100 Espinardo, Murcia, Spain; celiamaria.iniestaa@um.es (C.I.); rgbarrio@um.es (R.G.-B.); vanesa.nunez@um.es (V.N.-G.); mjperi@um.es (M.J.P.); fjgarcia@um.es (F.J.G.-A.)

**Keywords:** *Lycopersicon esculentum*, *Solanum lycopersicum*, carotenoids, lycopene, light-treatment, ultraviolet, lipophilic, antioxidant, ripening

## Abstract

Different strategies have been developed to increase the concentration of bioactive compounds in tomatoes during post-harvest, with ultraviolet light (UV) and light emitting diodes (LEDs) being interesting tools. The aim of this study was to evaluate the effect of ultraviolet (UVA at 366 nm and UVC at 254 nm) pre-treatment (1 kJ/m^2^) and red–blue LED light (25.4 µmol/m^2^/s) on the concentration of carotenoids, (poly)phenols and hydrophilic/lipophilic antioxidant capacity during 7 days of refrigeration storage of green tomatoes (*Solanum lycopersicum* L.) cultivar “Raf”. In addition, special attention was paid to quality parameters (weight loss, colour, acidity, soluble solids and ripening index). Tomatoes exposed to LED light at 6 °C for 7 days increased up to three times the total carotenoids content (mainly β-carotene and *E*-lycopene) compared to tomatoes refrigerated in the dark, while UV treatments alone did not significantly affect the carotenoid content. Besides, exposure to LEDs increased the hydrophilic and lipophilic antioxidant capacity of tomatoes by 30%, without affecting phenolic contents. Thus, LED treatments alone during refrigerated storage fostered ripening and improved the nutritional value of tomatoes, without compromising quality parameters. Further studies must be carried out to evaluate the impact on sensory attributes and consumer acceptance.

## 1. Introduction

The tomato (*Solanum lycopersicum* L.) is one of the most consumed fruits in the world. The Spanish cultivar “Raf” is highly desired by consumers due to its sweet flavour and crunchy and juicy texture. Intake of tomato has been inversely related to the incidence of cancer, cardiovascular diseases, ageing and many other health problems [1]. These beneficial effects of tomatoes are associated to their content in bioactive compounds, including antioxidants such as carotenoids (mainly lycopene), ascorbic acid, tocopherol, and phenolic compounds (mainly hydroxycinnamic acid derivatives and flavonols) [2,3]. The enhancement of bioactive compounds in tomatoes could influence food choices by consumers looking for health-promoting foods; besides, tomatoes rich in bioactive phytochemicals could be the raw material to develop functional foods, nutraceuticals or natural preservatives (such as antioxidant or antimicrobial agents) [4,5]. Since tomato is a climacteric fruit and continues to ripen after harvest, different strategies have been developed to increase the concentration of bioactive compounds during post-harvest storage and to maintain or enhance the quality during the shelf-life. Among them, treatments with ultraviolet light (UV) and light emitting diodes (LEDs)—alone or in combination—have been shown to be successful for this purpose, as light is an important regulator of the expression of carotenoid and (poly)phenols biosynthetic genes [6]. For instance, exposure to UVC (1–2 kJ/m^2^) prior storage increased the content of phenolic compounds, antioxidant capacity and lycopene contents in tomatoes at the “breaker stage” [7,8]. Additionally, exposure to LED light (64–113 μmol/m^2^/s) alone [9,10] or in combination with UV at room temperature [11] has been shown to be effective in enhancing the content of carotenoids, phenolic compounds, as well as the antioxidant capacity of tomatoes. These effects have been reported in mature green tomatoes stored at temperatures ranging from 14 to 22 °C, so we hypothesize that positive effects may be also obtained under refrigerated storage. Low temperatures of storage (<13 °C) have shown to delay ripening and decrease the biosynthesis of bioactive compounds [12], however, its combination with light treatments may enhance the content of carotenoids and phenolic compounds, as well as antioxidant activity, without showing a decline in other quality parameters, such as weight loss and the development of red colour. Therefore, the main aim of the present study was to ascertain whether the exposure of tomatoes from the Spanish cultivar “Raf”, harvested at a green stage, to red–blue LED light (25.4 µmol/m^2^/s) alone or in combination with UV light pre-treatment (UVA at 366 nm and UVC at 254 nm), could enhance the carotenoid content, total phenolic compounds and hydrophilic and lipophilic antioxidant activity during 7 days of cold storage at 6 °C. In addition, attention has been paid to other quality parameters such as colour, weight loss, titratable acidity, soluble solids and ripening index with the aim to evaluate characteristic changes affected by ripening and the overall quality of tomatoes exposed to different light treatments.

## 2. Results

### 2.1. Carotenoids Contents

The total contents of carotenoids after 7 days of storage were slightly increased as a consequence of ripening from 4.23 to 6.93 mg kg^−1^ (Table 1). As a result of LED treatments during storage, alone or in combination with UVA and UVC light pre-treatments, the total carotenoids content increased by three-fold with respect to control samples (7 days in darkness), as it is shown in Table 1. Among individual carotenoids, *E*-lycopene (5.5-fold) and all *Z*-lycopene isomers (three-fold) were effectively affected by LED exposure, with 15-*Z*-lycopene being the predominant isomer. Regarding β-carotene, significant increases were also observed after LED treatment (2.3-fold), with these effects being lower compared to lycopene.

No significant enhanced effects were observed when tomatoes were pre-treated with UVC and UVA and combined with LED light during storage. Similarly, UV pre-treated samples stored in the dark presented carotenoid concentrations that did not differ from controls at day 7. Thus, UV light alone failed to increase the carotenoids content in this experiment.

### 2.2. Total Phenolic Contents and Antioxidant Ccapacities

The content of total (poly)phenols in tomatoes after 7 days of storage were similar to the green tomatoes at day 0 (Table 2). Exposure to UV pre-treatments and LED light during 7 days of storage did not affect phenolic compounds, as no significant differences among samples were found, with the total phenolic compounds contents at the end of the experiment being similar to that of the initial green tomatoes (156–223 mg gallic acid equivalents (GAEs) kg^−1^).

Regarding the antioxidant capacity of tomatoes stored over different light conditions, both the hydrophilic (FRAP-H) and lipophilic (FRAP-L) antioxidant capacities increased by about 30% (1.5-fold) in samples stored under LED light alone or in combination with UVA and C pre-treatments (Table 2). Again, UV light pre-treatment alone failed to significantly enhance the antioxidant capacity of tomatoes, except for the UVA light pre-treatment that significantly increased the FRAP-H. Besides, no statistical differences were found among control samples at day 0 (green tomatoes) and samples stored during 7 days in the dark. Therefore, the natural process of ripening in cooling conditions (6 ± 1 °C; relative humidity 90 ± 1%) did not produce changes in the antioxidant capacity of tomatoes during storage, with the treatment with LED light being crucial to observe changes in this parameter.

Values of hydrophilic antioxidant capacity (ranged from 1602 to 2529 µmol trolox equivalents (TEs) kg^−1^) were higher compared to the lipophilic antioxidant capacity data (ranged from 128 to 222 µmol TEs kg^−1^), representing the hydrophilic antioxidant capacity, 92% of the total antioxidant capacity in every sample. Despite the fact that total phenolic compounds remained unchanged after light treatments, the antioxidant capacity was enhanced with LED light, indicating that other antioxidant compounds, such as vitamin C, might be accumulated in the fruits during storage, as we have seen with carotenoids.

### 2.3. Colour, Ripening Index and Weight Loss

With tomato ripening, carotenoids increased, and colour parameters shifted to more reddish values, with the colour parameters a* and Hue angle being the most affected, as it is shown in Table 3. Once again, LED treatments produced the highest changes in tomatoes colour, the a* parameter showed a three-fold increase (towards red colour) and the Hue angle dropped by half (from 107 to 50), also representing the change from the green to red colour. Besides, the lightness (L*) showed a reduction from day 0 to day 7 under all storage conditions, indicating the darkening of the red colour and being higher in the case of LED treatments. The ratio of colour variation a*/b* highly increased with ripening, indicating redness, being three-fold higher in tomatoes subjected to LED treatment alone or in combination with UV light. Finally, the increase in the overall colour difference (ΔE) confirmed LED treatments as majorly responsible for colour changes during the ripening of tomatoes, compared to control tomatoes stored in the dark.

**Figure 1:**
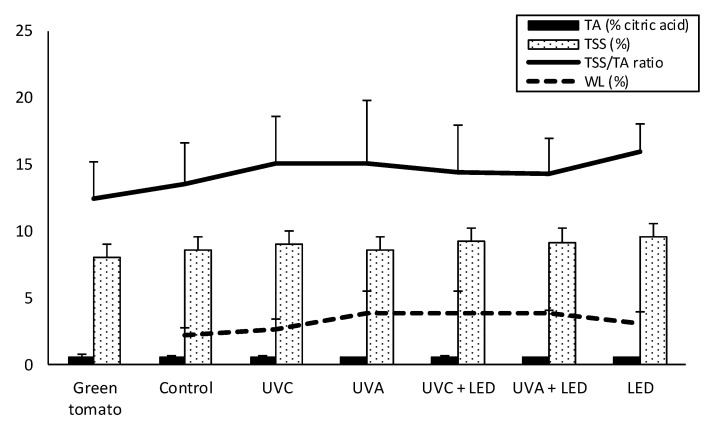
Changes in the ripening index (TSS/TA ratio) and related parameters of tomatoes exposed to different light treatments after 7 days of cold storage. Results are presented as mean values ± standard deviation (*n* = 3). TSS, total soluble solids; TA, titratable acidity; WL, weight loss.

As illustrated in Figure 1, light treatments weakly increased the content of TSS (total soluble solids), TA (titratable acidity) and the ripening index (TSS/TA ratio) in comparison to the control, mainly by slightly increasing TSS rather than by reducing acidity (TA). TSS were comprised in the range 8% to 10%, with the highest records in LED-treated samples, while TA remained unchanged (average value of 0.62%).

Accordingly, the TSS/TA ratio increased from 12 in green tomatoes (day 0) to an average of 15 in LED-treated samples. However, these changes did not achieve statistical significance. The weight loss ranged between 2.5% (samples stored in the dark) and 4% (UV + LED combined treatments); however, differences were not statistically significant.

## 3. Discussion

Considerable work has been performed to increase bioactive compound levels in tomatoes during post-harvest storage, with light treatments being a useful non-chemical tool for maintaining the overall quality and enhancing bioactivities of this fruit [13]. In our study, we tested the effect of combined UV (A or C) and red–blue LED light treatments on the concentration of carotenoids, total phenolic compounds and antioxidant capacities (from hydrophilic and lipophilic nature) of mature green tomatoes stored for 7 days under refrigeration conditions. Our results showed that the key condition to increase carotenoid contents and hydrophilic and lipophilic antioxidant capacities was the exposure to continuous LED light, whilst the effect of UV pre-treatments alone was negligible. In this context, it has been shown that red and blue LED lights are able to modulate the expression of light receptors and several genes involved in carotenoid biosynthesis pathways, influencing the nutritional value of fruits and vegetables and being beneficial for human health [14,15].

The effectiveness of LED light and UV treatments to enhance carotenoid synthesis has been previously reported in tomatoes stored at room temperature, showing a 1.5-fold increase in these compounds with LED light, alone or in combination with UV light [11]. Intermittent exposure (1 h/day) to a high red to far red light ratio at 19 °C also resulted in a 41% increase in lycopene concentration after 6 days of storage [9]. In line with our results, the continuous exposure of tomatoes to red LED light under a controlled temperature (19–20 °C) resulted in an approximately six-fold increase in lycopene content after 10 days of storage [10]. Interestingly, we have achieved a comparable yield in total lycopene (4.5-fold) and total carotenoids (five-fold) during our shorter refrigeration storage trial (7 days). That means that our cold temperature (6 °C) of storage allowed the biosynthesis of carotenoids, although it is well known that refrigerated storage slows the metabolic activity of tomatoes, respiration rate, transpiration, ethylene production, and thus delayed ripening and senescence [16,17]. In a previous work, light-red tomatoes under refrigerated storage (7 °C) inhibited lycopene accumulation in comparison to tomatoes stored above 15 °C, that yielded a three-fold higher lycopene content [12]. Accordingly, we also observed slowed lycopene accumulation in our control samples stored in darkness. Importantly, this effect was counteracted by exposure to LED without the overheating of tomatoes due to lamp irradiation (temperature in the LED chamber never exceeded 7 °C). This might partly explain why, in a shorter time, we reached similar increases in total lycopene than those obtained at higher storage temperatures by other authors [9,10,11].

On the other hand, exposure to either UV or LED light had no effect on total phenolic compounds, despite the fact that both light treatments are known to modulate the expression of an array of genes involved in the biosynthesis of phenolic compounds and carotenoids [14,18]. In regard to UV light, our data are in accordance with previous reports. For instance, no changes in total phenolic compounds were reported in mature green tomatoes pre-exposed to UVC (2 kJ/m^2^) and stored for 7 days at 14 °C [19] or pre-treated with UVC (1 kJ/m^2^) and stored for 8 days at room temperature [7]. A similar behaviour was reported by Panjai et al. [11], who applied a higher UV intensity (5 kJ/m^2^) but in short daily exposures (30 min every day) for 20 days and did not find differences in total phenolic compounds. However, higher UV intensities (8–12 kJ/m^2^) and/or longer storage time (>7 days) were needed to achieve significant increases in total phenolic compounds [7,19]. Thus, the lack of effect of UV light on total phenolic compounds in our study might be attributable to the low light intensity applied (1 kJ/m^2^) and/or to the short duration of the storage trial (7 days). Besides, a role for refrigeration to prevent UV from triggering further phenolic compounds biosynthesis during storage cannot be precluded. Moreover, one should bear in mind that the effect of UV irradiation treatments in tomatoes is cultivar-dependent and dissimilar effects have been reported by other authors [18].

Our data also showed that exposure to LED light was not able to increase total phenolic compounds. This is in contrast with previous studies that reported significant increases in total phenolic compounds in LED-treated tomatoes stored from 5 to 10 days under a controlled room temperature (19–20 °C) [10,11]. However, our results are more in line with those reported in tomatoes exposed to yellow LED light and stored for 7 days at a lower temperature (10 °C) [20]. Yellow LEDs are also known to enhance the ripening of fruits along with induction of the synthesis of antioxidant compounds, including (poly)phenols and carotenoids [15]. However, Kokalj et al. [20] observed that, in comparison to the initial samples, total phenolic compounds remained unchanged in LED-treated tomatoes, and even decreased in control samples stored in darkness. These data suggest that the lower the storage temperature, the weaker the effect of LED irradiation on phenolic compounds. For this reason, we hypothesise that the lack of effect of our LED light conditions on phenolic compounds might partly be due to low storage temperatures. Concerning this, it has been shown that the refrigerated storage of fruits and vegetables tend to reduce their total phenolic content, particularly by 50% in tomato [21]. These authors proposed that the decreasing trend observed in total phenolic compounds could be due to degradation processes, following a change in the pattern of related-enzymes activity, such as a decrease in phenylalanine ammonia-lyase (PAL) or increase in polyphenoloxidase (PPO) under refrigeration. Accordingly, it was shown that refrigeration at 4 °C induced oxidative stress in tomato tissues, increasing reactive oxygen species (ROS) and suppressing antioxidant enzymes [22]. Thus, it is reasonable to think that under our experimental conditions (6 ± 1 °C for 7 days) (poly)phenols might have been acted as sacrificial antioxidants to counteract increased oxidative stress. In this way, any expected light-induced increase in total phenolic compounds might have been masked due to (poly)phenol consumption for ROS scavenging.

Regarding antioxidant capacity, although the ferric reducing power (FRAP) method was initially developed to evaluate the hydrophilic antioxidant activity of samples, Müller et al. [23] modified the classical assay to develop a new FRAP method using *n*-hexane to dissolve the lipophilic antioxidant of the samples, allowing the measurement of lipophilic components of samples, such as carotenoids and vitamin E [23]. Our results showed that both hydrophilic and lipophilic antioxidant properties increased (1.7-fold) with LED light treatments, exhibiting the hydrophilic fraction as 92% of the total antioxidant capacity, as reported by other authors [24,25,26]. According to our results, similar increases in the antioxidant capacity of tomatoes during post-harvest storage at a low temperature (5 °C) have been described, with this temperature being more effective compared to higher temperatures of storage [27]. Despite this increase, phenolic compounds remained unchanged, with these compounds being highly related with the hydrophilic antioxidant capacity in fruits and vegetables. Such a behaviour has been previously reported [7,19,24], meaning that other compounds not determined in our study (e.g., ascorbic acid) might have been responsible for this enhanced hydrophilic antioxidant capacity. In this sense, ascorbic acid levels increased under LEDs by about two-fold in mature green tomatoes kept at 18 °C, in comparison to darkness storage [28]. Nevertheless, the possibility of carotenoids being extracted to some extent in methanol 80%, the extraction solvent for the hydrophilic fraction evaluation, should not be ruled out, although lycopene and β-carotene are nearly insoluble in methanol [29].

In our study, the increase in the lipophilic antioxidant capacity was significantly positively correlated with the increase in lycopene with ripening (r = 0.88; *p* < 0.001), as this carotenoid has been described as a lipophilic antioxidant highly active against free radicals and the most important antioxidant in the organic phase [25]. In addition, β-carotene content has also exhibited a significant positive association with lipophilic antioxidant capacity (r = 0.86; *p* < 0.001), having strong antioxidant potential [30]. Thus, the enhancement of carotenoids contents in tomato using LEDs during post-harvest storage, especially lycopene, is of great interest in the marketing of tomato, which looks for the development of high pigment varieties containing natural antioxidants by the use of different tools, such as breeding programmes [31].

Last but not least, the pattern of changes observed for quality parameters agreed with previous reports [9,32,33]. Colour parameters related with ripening, such as the increase in a* and decrease in Hue angle, changed in a similar way as described by other authors [9,32] and paralleled the changes found in carotenoids. The sharp increase in red colour pigments, such as carotenoids, were reported between stages 2 and 5 of ripening (breaker to light red), together with a decrease in the L* value [33]. Therefore, the increase in the ratio of colour variation a*/b* and the overall colour difference (ΔE), both related with redness, showed a strong positive correlation with the total lycopene content (r > 0.88; *p* < 0.001), and thus, with total carotenoids (r > 0.85; *p* < 0.001).

On the other hand, although no significant differences were found in TSS (8–10%) and TA (0.58–0.65%) in the tomatoes after 7 days of storage, the slight trend to increase observed for the ripening index (TSS/TA ratio) was consistent with other authors [9,11]. Although this ratio is currently used as a ripening index, it does not always correlate with flavour perception, as different concentrations of TSS and TA can provide the same ratio and, especially in tomato, TSS highly depends of the medium-size of the fruit [34]. Refrigeration temperatures might well explain why TSS, TA, and consequently the TSS/TA ratio, barely changed during storage. During ripening, the increment of TSS is caused by biosynthetic processes or the degradation of polysaccharides, whilst the amount of organic acid usually decreases because it is substrate of respiration [17]. Therefore, cooling storage slows the metabolic activity and rate of transpiration and delays the usual shift in soluble solids and organic acids, and therefore changes in the taste of tomatoes during storage. Weight loss remained practically unchanged in our study and was also in line with results from Sualeh et al. [17], who reported higher TA, lower TSS and lower weight losses in refrigerated tomatoes versus controls at room temperature.

**Figure 2:**
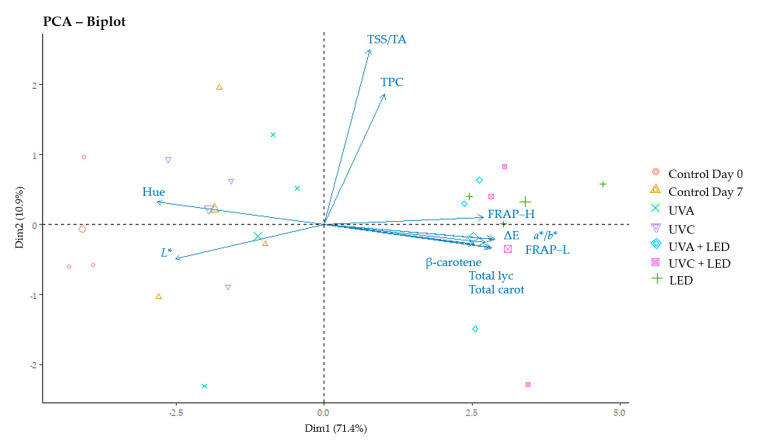
Biplot analysis for changes in the main representative variables of the study. Hue, hue angle; L*, lightness; TSS/TA, ratio of ripening index; TPC, total phenolic compounds; FRAP−H, hydrophilic antioxidant capacity; FRAP−L, lipophilic antioxidant capacity; ΔE, overall colour difference; a*/b*, ratio of colour variation; total lyc, total lycopene; total carot, total carotenoids. PCA: principal component analysis.

Finally, we performed a principal component analysis (PCA) to visualize the relationship among the variables understudied and the different light treatments performed during 7 days of cold storage. Among all parameters analysed, we selected 11 variables (hue angle, lightness, ratio of ripening index TSS/TA, total phenolic compounds, hydrophilic antioxidant capacity, lipophilic antioxidant capacity, overall colour difference ΔE, ratio of colour variation a*/b*, β-carotene, total lycopene and total carotenoids) as the most meaningful of the study (Figure 2), since the remaining parameters examined, such as individual carotenoids and the parameters of colour a*, b* and Chrome, did not offer additional information. The PCA identified 11 principal components (PCs), the first two explaining the 82.3% of the total variance (R^2^). In PC1 (R^2^ = 71.4%), the colour parameters hue angle and L* were negatively associated, while the variables related to the antioxidant capacity (FRAP-H and FRAP-L), carotenoids (β-carotene, total lycopene and total carotenoids), and colour changes (ΔE and ratio a*/b*) were positively associated. In PC2 (R^2^ = 10.9%), the variables TSS/TA and TPC were positively related. Figure 2 clearly differentiates between LEDs treatments, finding these samples on the right side of PC1 and near the trait vectors related to redness and the increase in carotenoids in tomatoes, and the other samples, controls and UV treatments, which were found in the left side of PC1. Control samples at day 0 were found farthest from the previous cited variables and near the colour parameters related to green tomatoes (hue angle and L*).

Pre-treatment with UVA light alone seemed to have a higher positive impact on carotenoids, antioxidant capacity and redness of tomatoes compared to UVC. On the contrary, when UV pre-treatments were combined with LEDs, UVC seemed to have a greater influence on carotenoids and FRAP-L. Nevertheless, these effects were not statistically significant. The PCA biplot showed clearly that LED light treatments can be a successful tool to enhance the carotenoids content, antioxidant capacity and redness of tomatoes under refrigeration temperatures for 7 days, and confirm a slight influence of light treatments on the variables TPC and TSS/TA ripening ratio.

Regarding energy consumption of the light treatments, UV pre-treatments consumed 0.01 kWh, with 0.05 kW being the pre-treatment for 5 h. On the other hand, LED lights consumed 0.02 kWh, being 3.36 kW for 7 days of treatment of tomatoes during storage. Thus, the use of UV and/or LED lights (3.41 kW in total) involved significant reductions in energy consumption compared to traditional incandescent light bulbs, which have been reported by the U.S. Department of Energy to consume a minimum of 75% more energy, being the average consumption of a 40 W bulb of 0.4 kWh [35].

## 4. Materials and Methods

### 4.1. Reagents and Chemicals

Carotenoid standards (lycopene and β-carotene), gallic acid, Folin–Ciocalteu’s phenol reagent and 6-hydroxy-2,5,7,8-tetramethylchroman 2-carboxylic acid (Trolox) were purchased from Sigma–Aldrich (St. Louis, MO, USA). Ethanol, methanol, acetone, hexane and tert-butyl methyl ether were purchased from Merck KGaA (Darmstadt, Germany), 2,4,6-tripyridyl-s-triazine (TPTZ) was purchased from Fluka Chemie GmbH (Buchs, Switzerland). All other reagents were of the highest grade obtainable.

### 4.2. Samples and Experimental Design

Tomatoes (*Solanum lycopersicum* L.) cultivar “Raf” (Cañada Natural S.A., Andalucía, Spain), belonging to the same batch, were purchased at breaker ripening stage. The breaker stage is featured by a definite break in colour from green to tannish-yellow, pink or red on no more than 10 percent of the surface [36]. For the experiments, tomatoes were washed, randomly assigned to each experimental condition (three tomatoes per condition) and weighted prior to light treatments. Three independent experiments were performed, and prior to analysis the three tomatoes per experiment were mixed, obtaining three samples overall (*n* = 3).

### 4.3. Light Conditions

**Figure 3:**
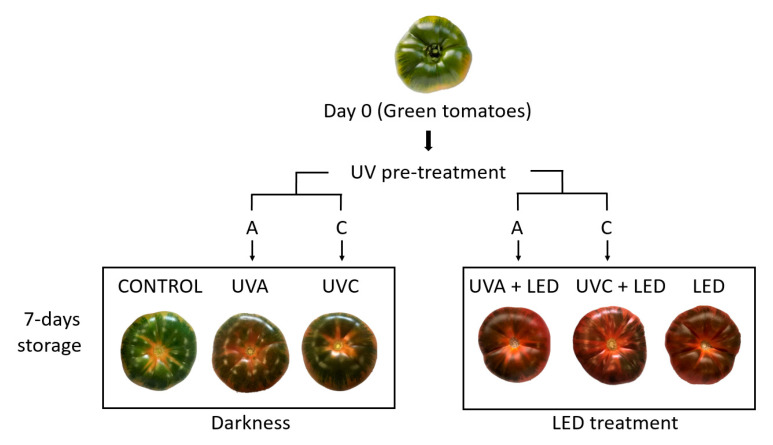
Light conditions used for the refrigerated storage of tomatoes.

Pre-treatments with UVA and UVC lasted 5 h; after 2.5 h tomatoes were turned over to ensure the UV light exposure of both fruit sides. Light treatments (LED and darkness) lasted 7 days and were performed under refrigeration (6 ± 1 °C; relative humidity 90 ± 1%). For this experiment, six different light treatments were used (Figure 3):

1. CONTROL: Untreated control samples stored in darkness;
2. UVA: Samples pre-treated with ultraviolet light-A and later stored in darkness;
3. UVC: Samples pre-treated with ultraviolet light-C and later stored in darkness;
4. UVA + LED: Samples pre-treated with ultraviolet light-A and later stored under continuous red-blue light;
5. UVC + LED: Samples pre-treated with ultraviolet light-C and later stored under continuous red-blue light;
6. LED: Samples stored under continuous red-blue light.

**Figure 4:**
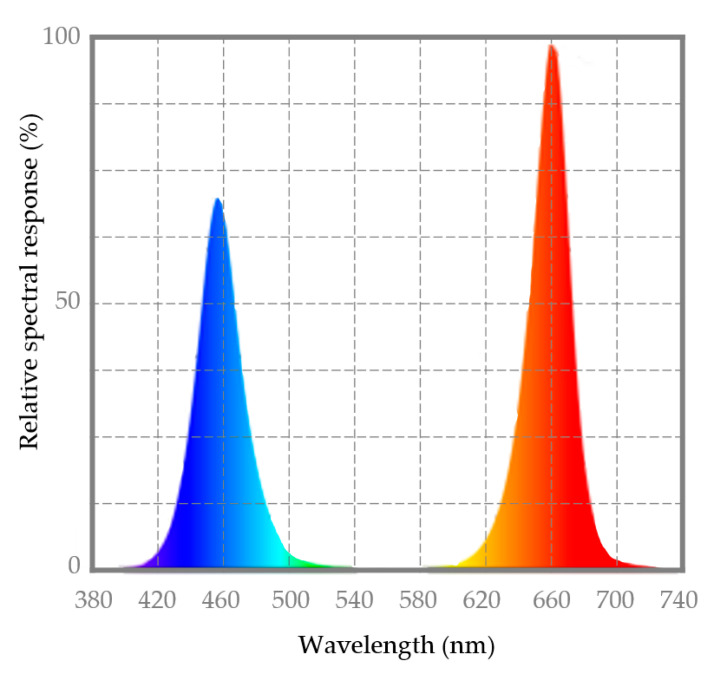
LED light spectra of a lamp with 9 red diodes (λ = 620–625 nm) and 3 blue diodes (λ = 460–467 nm).

The UV-light pre-treatment (1 kJ/m^2^) was performed at room temperature (20 ± 1 °C; relative humidity 63 ± 3%) in an UV-viewing cabinet (Panreac) equipped with two 8 W lamps emitting UVA (λ = 366 nm) and UVC (λ = 254 nm) [7]. The LED treatment during storage was carried out using a 24 W LED lamp (Light K5, Kmashi, WNT-LUXTECH CO., Ltd.) equipped with 9 red diodes (λ = 620–625 nm) and 3 blue diodes (λ = 460–467 nm). Light intensity in the treatment area (3.7 kLux) was measured using a digital light meter (ILM 1335, ISO-TECH) and photosynthetic photon flux density (PPDF) (25.4 µmol/m^2^/s) and light spectra (Figure 4) were measured using a spectrophotometer ALP-01 (Asensetek Incorporation, New Tai Pai City, Taiwan). The data of the energy consumption of the different light treatments were measured using a Wi-Fi smart plug with energy monitoring EG-EW003MC (Energeeks Iberia S.L., Madrid, Spain).

In order to ensure light exposure of both fruit sides, tomatoes were turned over daily. After the treatments, samples were homogenised and kept frozen at −20 °C until analysis. Green tomato samples (*n* = 3) were also analysed as the initial control (day 0). The results were expressed as the mean ± SD of three independent experiments.

### 4.4. Analysis of Carotenoids by HPLC-DAD

Carotenoids were analysed by high-performance liquid chromatography with diode array detector (HPLC-DAD) according to Böhm (2001) [37] with some modifications. For carotenoid extraction, 0.25 g of fresh tomato was mixed with 10 mL of hexane:acetone:ethanol (2:1:1, *v*/*v*) in 50 mL tests tubes Falcon^®^, vortexed for 10 s, and then sonicated using an ultrasound bath (Branson 5510, 135 W, 42 kHz, Eemnes, The Netherlands) for 5 min. After that, 2 mL of distilled water was added, the resulting mixture was vortexed again for 10 s, and allowed to stand for 15 min to facilitate phase separation. One millilitre of the non-polar phase containing the carotenoids (upper-layer) was transferred to a glass test tube and evaporated under nitrogen stream. The residue was redissolved in 1 mL of tert-butyl methyl ether:methanol (1:1, *v*:*v*), centrifuged at 20,800× *g* for 10 min at room temperature and the supernatant obtained was injected in the HPLC system.

The analyses were performed in an Agilent 1200 HPLC system, fitted with a quaternary pump, a degasser, a thermostatted column support, an autosampler, and a serial diode detector (Agilent Technologies, Spain). Chromatography separation was carried out using a C30 column 250 mm × 4.6 mm, 5 µm i.d. (Análisis Vínicos S.L., Villarobledo, Spain) maintained at 17 °C, and using tert-butyl methyl ether (A) and methanol (B) as mobile phases at a flow of 1 mL/min. The gradient started with 2% A in B to reach 35% A at 35 min, 60% A at 45 min, 60% at 55 min and returning to the initial conditions (2% A in B) for 5 min before the next injection. Carotenoids were identified according to their ultraviolet spectra and retention times by chromatographic comparisons with authentic standards. The compound β-carotene was quantified at 450 nm using β-carotene as the standard, whilst *E*-lycopene and its *Z*-isomers were quantified at 472 nm using *E*-lycopene as the standard. As standards of lycopene *Z*-isomers were not available, they were tentatively identified based on their retention times and spectral characteristics described in the literature [38,39]. The results were expressed as mg carotenoids per kg fresh weight of tomato.

### 4.5. Analysis of Total Phenolic Compounds

The total phenolic compounds content was quantified by a colorimetric assay using Folin–Ciocalteu’s phenol reagent [40]. For sample extraction, 1 g of fresh tomato was extracted with 10 mL of 80% (*v*/*v*) aqueous methanol containing 1% HCl in a 50 mL test tube Falcon^®^. After mixing for 10 s in a vortex mixer (Wisd VM-10 model, Witeg Labortechnik GmbH, Wertheim, Germany), the extraction was performed by sonication in an ultrasound bath (Branson 5510, 135 W, 42 kHz, Branson Ultrasonics B.V., Eemnes, Netherlands) for 25 min. Then, extracts were centrifuged at 4500× *g* for 10 min and aliquots of the supernatant were used for the analyses. For the analysis of total phenolic compounds, 500 μL of 0.2 N Folin–Ciocalteu’s phenol reagent and 400 μL of a 2 M Na_2_CO_3_ solution were added to 100 μL of tomato extract. After incubation for 2 h in darkness at room temperature, absorbance at 750 nm was measured against water as a blank in a spectrophotometer (Evolution 300, Thermo scientific, Cambridge, UK). Gallic acid in the range 0 to 100 mg L^−1^ was used for linear calibration (R^2^ = 0.99), and the total phenolic content in the samples was expressed as mg of gallic acid equivalents (GAEs) per kg fresh weight of tomato.

### 4.6. Analysis of Hydrophilic and Lypophilic Antioxidant Capacity

The total antioxidant capacity was measured by the application of the ferric reducing power (FRAP) assay [41] for both hydrophilic and lipophilic antioxidants. For the hydrophilic compounds, the extraction for phenolic compounds described above (Section 4.5) was used. Then, 100 μL of the extract was mixed with 1 mL of FRAP reagent and absorbance readings at 593 nm were taken after 4 min of incubation. The FRAP reagent was freshly prepared by mixing a 10 mM TPTZ solution in 40 mM HCl, 20 mM FeCl_3_·6H_2_O solution and 0.3 M acetate buffer (pH 3.6) in the ratio of 1:1:10. For the lipophilic compounds, the previously described carotenoid extraction method (Section 4.4) was used. Subsequently, the lipophilic FRAP method described by Müller et al., [23] with some modifications was performed. Briefly, 300 µL of the hexane phase were mixed with 1 mL of the prepared FRAP reagent for 4 min using a vortex mixer (Wisd VM-10 model, Witeg Labortechnik GmbH, Wertheim, Germany). Then, the solutions were centrifuged (4500× *g*, 2 min) to separate the phases and the absorbance of the lower phase was measured at 593 nm in a spectrophotometer (Evolution 300, Thermo scientific, Cambridge, UK). Trolox was used as standard in the range of 10–250 μM (equation correlation coefficient R^2^ = 0.99), using water or hexane as blanks for the hydrophilic or lipophilic methods, respectively. The results were expressed as μmol Trolox equivalents (TE) per kg fresh weight of tomato.

### 4.7. Colour Measurements, Ripening Index and Weight Loss

Colour (CIELab space) was measured in homogenised tomato samples using a colorimeter (Chroma meter CR300, Konika-Minolta Business Solutions Europe GmbH, Langenhagen, Germany) and reported as L*, a* and b*, Chroma and Hue values. The L* value represents lightness, ranging from 0 (black) to 100 (white), and indicates how dark/light the sample is; the a* value exhibits redness (positive) and greenness (negative); and the b* value exhibits yellowness (positive) and blueness (negative). The colour index was calculated as the a*/b* ratio [9]. The total colour difference (ΔE) was used to characterize the overall change in colour during the storage and was calculated by the following Equation (1) [42]:

(1) $$\Delta E_{ab}^{*}=\;\sqrt{{\left(L_{2}^{*}-L_{1}^{*}\right)}^{2}+\;{\left(a_{2}^{*}-a_{1}^{*}\right)}^{2}+\;{\left(b_{2}^{*}-b_{1}^{*}\right)}^{2}}$$

where $L_{1}^{*}$, $a_{1}^{*}$ and $b_{1}^{*}$ are values of green tomatoes at day 0 (initial control).

The ripening index was calculated as the total soluble solids (TSS) to titratable acidity (TA) ratio [43]. The results of TSS were expressed as % and were measured at 20 °C using a digital refractometer (Abbemat 200, Anton Paar GmbH, Graz, Austria). Titratable acidity, expressed as % citric acid, was determined by titrating with NaOH (0.1 N) up to pH 8.1 according to the Association of Official Analytical Chemists (AOAC) official method 942.15 [44]. Finally, weight loss was calculated by differences between the initial weight (day 0) and final weight divided by initial weight, expressed as %.

### 4.8. Statistical Analyses

The results from three different experiments (*n* = 3) were analysed by IBM-SPSS 24.0 software package (IBM, Armonk, NY, USA). Comparisons between means were analysed by one-way analysis of variance (ANOVA) followed by the least significant difference (LSD) test. Pearson’s correlation test was performed to study relationships between variables, *p* values < 0.05 were considered statistically significant. The Biplot analysis was performed using R studio, version 3.4.3 (R Foundation for Statistical Computing, Vienna, Austria).

## 5. Conclusions

Our data established that refrigerated storage under continuous red–blue LED light for 7 days improves the nutritional value of tomatoes by increasing the total carotenoid content and total antioxidant capacity, without losing (poly)phenols. Besides, no significant changes in the ripening index and weight loss indicated the quality maintenance of tomatoes during storage. LED light alone was enough to elicit a significant increase in carotenoid contents in comparison with a combination of UV pre-treatment and LED light. From a technological point of view, this can be considered an advantage, since is not necessary to install UV lamps in addition to the safe and non-thermal LED lights to achieve an enhanced effect on carotenoid accumulation. Additionally, LEDs are more energy-efficient than other traditional lights, such as incandescent bulbs. Further studies must be carried out to evaluate the impact of this light treatment on sensory attributes and consumer acceptance.

## Figures and Tables

**Table 1:** Carotenoid content (mg kg^−1^) of green tomato (control at day 0) and tomatoes exposed to different light treatments for 7 days in refrigeration conditions. Mean values ± standard deviation from samples from three different experiments (*n* = 3). UV: ultraviolet light; LED: light emitting diode.

Light Source	β- Carotene	*E*- Lycopene	15-*Z*- Lycopene	13-*Z*- Lycopene	9-*Z*- Lycopene	Total *Z*- Lycopene	Total Lycopene	Total Carotenoids
Day 0	3.23 ± 0.30 ^d^	0.57 ± 0.06 ^b^	0.15 ± 0.01 ^b^	0.15 ± 0.01 ^b^	0.14 ± 0.01 ^c^	0.44 ± 0.0 ^b^	1.00 ± 0.07 ^b^	4.23 ± 0.33 ^b^
Day 7								
Control	3.95 ± 0.78 ^c,d^	2.25 ± 0.78 ^b^	0.34 ± 0.10 ^b^	0.19 ± 0.04 ^b^	0.21 ± 0.05 ^b,c^	0.73 ± 0.16 ^b^	2.98 ± 0.93 ^b^	6.93 ±1.65 ^b^
UVA	4.91 ± 1.55 ^b,c,d^	2.59 ± 0.19 ^b^	0.32 ± 0.01 ^b^	0.18 ± 0.02 ^b^	0.20 ± 0.07 ^b,c^	0.70 ± 0.08 ^b^	3.28 ± 0.27 ^b^	8.19 ±1.82 ^b^
UVC	3.43 ± 1.28 ^d^	1.64 ± 0.68 ^b^	0.26 ± 0.06 ^b^	0.17 ± 0.01 ^b^	0.17 ± 0.01 ^b,c^	0.60 ± 0.06 ^b^	2.24 ± 0.74 ^b^	5.68 ±2.02 ^b^
UVA + LED	5.81 ± 0.19 ^a,b,c^	8.90 ± 1.13 ^a^	1.11 ± 0.11 ^a^	0.35 ± 0.08 ^a^	0.40 ± 0.1 ^a^	1.85 ± 0.38 ^a^	10.75 ± 1.51 ^a^	16.57 ± 1.70 ^a^
UVC + LED	7.42 ± 1.31 ^a^	11.27 ± 2.85 ^a^	1.42 ± 0.34 ^a^	0.34 ± 0.02 ^a^	0.32 ± 0.02 ^a,b^	2.08 ± 0.39 ^a^	13.35 ± 3.24 ^a^	20.77 ± 4.55 ^a^
LED	6.58 ± 1.09 ^a,b^	12.41 ± 6.05 ^a^	1.19 ± 0.61 ^a,b^	0.58 ± 0.32 ^a^	0.27 ± 0.07 ^a,b,c^	2.03 ± 1.00 ^a^	14.44 ± 7.05 ^a^	21.02 ± 8.14 ^a^

^a–d^ Different superscript letters within columns mean statistically significant differences at *p* < 0.05.

**Table 2:** Effect of different illumination conditions on total phenolic compounds (TPCs) and hydrophilic (FRAP-H) and lipophilic (FRAP-L) antioxidant activity of tomatoes during cold storage. Mean values ± standard deviation from samples from three different experiments (*n* = 3).

Light Source	TPCs (mg GAEs ^1^ kg^−1^)	FRAP-H (µmol TEs ^2^ kg^−1^)	FRAP-L (µmol TEs kg^−1^)
Day 0	193 ± 16	1602 ± 114 ^c^	128 ± 16 ^b^
Day 7			
Control	202 ± 78	1766 ± 183 ^b,c^	153 ± 2.3 ^b^
UVA	156 ± 58	1846 ± 87 ^b^	139 ± 21 ^b^
UVC	172 ± 13	1692 ± 114 ^b,c^	142 ± 9.3 ^b^
UVA + LED	206 ± 33	2529 ± 40 ^a^	198 ± 37 ^a^
UVC + LED	200 ± 65	2402 ± 120 ^a^	222 ± 11 ^a^
LED	223 ± 22	2446 ± 25 ^a^	214 ± 7.9 ^a^

^1^ GAE: gallic acid equivalents; ^2^ TEs: trolox equivalents. ^a–c^ Different superscript letters within columns mean statistically significant differences at *p* < 0.05.

**Table 3:** Colour parameters of tomatoes exposed to different light treatments during 7 days of cold storage. Mean values ± standard deviation from samples from three different experiments (*n* = 3).

Light Source	L*	a*	b*	a*/b*	Chroma	Hue	ΔE
Day 0	55.41 ± 4.08 ^a^	−7.02 ± 1.66 ^c^	23.07 ± 4.58 ^a^	−0.32 ± 0.14 ^c^	24.19 ± 4.03	107.71 ± 6.78 ^a^	
Day 7							
Control	49.77 ± 1.88 ^b^	2.21 ± 1.64 ^b^	21.22 ± 3.42 ^a,b^	0.11 ± 0.08 ^b^	21.39 ± 3.34	84.10 ± 4.86 ^b^	11.37± 2.34 ^b^
UVA	48.88 ± 2.59 ^b^	4.23 ± 3.86 ^b^	20.28 ± 3.43 ^a,b^	0.19 ± 0.16 ^b^	20.95 ± 4.10	79.98 ± 8.79 ^b^	14.07 ± 1.33 ^b^
UVC	48.63 ± 1.18 ^b,c^	2.48 ± 3.12 ^b^	19.66 ± 3.28 ^a,b^	0.11 ± 0.14 ^b^	20.00 ± 3.61	84.26 ± 7.97 ^b^	12.73 ± 0.92 ^b^
UVA + LED	46.35 ± 0.95 ^b,c^	14.12 ± 2.09 ^a^	15.66 ± 4.51 ^b^	0.93 ± 0.14 ^a^	21.14 ± 4.74	47.01 ± 4.20 ^c^	24.53 ± 0.31 ^a^
UVC + LED	44.68 ± 0.93 ^c^	12.36 ± 0.74 ^a^	15.83 ± 2.94 ^b^	0.79 ± 0.10 ^a^	20.12 ± 2.77	51.55 ± 3.55 ^c^	23.43 ± 0.15 ^a^
LED	44.86 ± 2.90 ^c^	12.92 ± 0.89 ^a^	15.97 ± 4.34 ^b^	0.85 ± 0.21 ^a^	20.63 ± 3.84	50.19 ± 6.61 ^c^	23.97 ± 2.08 ^a^

^a–c^ Different superscript letters within columns mean statistically significant differences at *p* < 0.05.

## Data Availability

The raw data (*n* = 3) of the mean values ± standard deviation presented in this study are available on request from the corresponding author.

## Sample Availability

Frozen samples of tomatoes will be available on request from the corresponding author for one year.
